# CAREGIVING STRESS IN ADRD CAREGIVERS: THE INFLUENCE OF SOCIAL DETERMINANTS OF HEALTH AND SOCIAL SUPPORT

**DOI:** 10.1093/geroni/igae098.3823

**Published:** 2024-12-31

**Authors:** Kyungmi Lee, Chunhong Xiao

**Affiliations:** Case Western Reserve University Frances Payne Bolt, Cleveland, Ohio, United States; University of Alabama at Birmingham/School of Nursing, Birmingham, Alabama, United States

## Abstract

Alzheimer’s disease and related dementias (ADRD) caregivers face significant physical, emotional, and financial challenges. These stressors are influenced by social determinants of health (SDOH), such as income and service accessibility, as well as caregiver-specific factors like health status and caregiving burden. Social support can help mitigate caregiving stress. This study aimed to examine the relationships among SDOH, caregiver-specific factors, social support, and caregiving stress in ADRD caregivers. Utilizing data from the Caregiving in the U.S. 2020 study (n = 357 ADRD caregivers), we conducted a multiple regression analysis (F = 15.236, p < 0.001). Results show that social support significantly reduces caregiving stress (β = -0.261, p = 0.004). Caregiver health status (β = -0.592, p < 0.001), difficulty accessing services (β = 0.770, p < 0.001), and caregiving burden (β = 0.511, p < 0.001) are also significant predictors of stress. Although age did not directly impact caregiving stress, it significantly influenced service access (β = -0.010, p = 0.036), which in turn affected stress levels. Financial strain (β = 0.270, p < 0.001) and physical strain (β = 0.171, p = 0.005) were also associated with service accessibility issues. These findings suggest that enhancing social support and improving service access could effectively reduce caregiving stress. Future research should further investigate these areas to develop targeted interventions for ADRD caregivers.

